# Current strategies and successes in engaging women in vector control: a systematic review

**DOI:** 10.1136/bmjgh-2017-000366

**Published:** 2018-01-07

**Authors:** Jayleen K L Gunn, Kacey C Ernst, Katherine E Center, Kristi Bischoff, Annabelle V Nuñez, Megan Huynh, Amanda Okello, Mary H Hayden

**Affiliations:** 1 Department of Epidemiology and Biostatistics, Mel and Enid Zuckerman College of Public Health, University of Arizona, Tucson, Arizona, USA; 2 Research Department, BrightOutcome, Inc, Buffalo Grove, Illinois, USA; 3 University of Arizona Health Sciences Library, Mel and Enid Zuckerman College of Public Health, University of Arizona, Tucson, Arizona, USA; 4 Research Applications Laboratory, National Center for Atmospheric Research, Boulder, Colorado, USA

**Keywords:** infections, diseases, disorders, injuries, systematic review, dengue, malaria, prevention strategies

## Abstract

**Introduction:**

Vector-borne diseases (VBDs) cause significant mortality and morbidity in low-income and middle-income countries and present a risk to high-income countries. Vector control programmes may confront social and cultural norms that impede their execution. Anecdotal evidence suggests that incorporating women in the design, delivery and adoption of health interventions increases acceptance and compliance. A better understanding of programmes that have attempted to increase women’s involvement in vector control could help shape best practices. The objective of this systematic review was to assess and critically summarise evidence regarding the effectiveness of women participating in vector control.

**Methods:**

Seven databases were searched from inception to 21 December 2015. Two investigators independently reviewed all titles and abstracts for relevant articles. Grey literature was searched by assessing websites that focus on international development and vector control.

**Results:**

In total, 23 articles representing 17 unique studies were included in this review. Studies discussed the involvement of women in the control of vectors for malaria (n=10), dengue (n=8), human African trypanosomiasis (n=3), schistosomiasis (n=1) and a combination (malaria and schistosomiasis, n=1). Seven programmes were found in the grey literature or through personal communications. Available literature indicates that women can be successfully engaged in vector control programmes and, when given the opportunity, they can create and sustain businesses that aim to decrease the burden of VBDs in their communities.

**Conclusion:**

This systematic review demonstrated that women can be successfully engaged in vector control programmes at the community level. However, rigorous comparative effectiveness studies need to be conducted.

**Key questions**

**What is already known about this topic?**

- Barriers exist for employing women in vector control as cultural norms may prohibit women from participating.
- In order to meet the Sustainable Development Goals 5 and 8, development programmes should provide equal access to work for both men and women.
- The scientific literature has called attention to the important role women may play in vector control, yet no published literature review has assessed strategies to engaging women in vector control.

**What are the new findings?**

- Available literature indicates that women can be successfully engaged in vector control programmes and, when given the opportunity, they can create and sustain businesses that aim to decrease the burden of vector-borne diseases in their communities.
- Both peer-reviewed literature and grey literature support the idea of including women in vector control programmes.
- When given the initial supplies, women can create a self-sustaining vector control programme.

**Recommendations for policy**

- Policies that support women’s participation in the vector control work force are needed.
- There is a significant evidence gap for the relative effectiveness of vector control in women-centred vector control programmes. Rigorous comparative effectiveness studies need to be conducted.

## Introduction

### Background

Despite concerted efforts to reduce vector-borne diseases (VBDs), they still represent a heavy burden in low-income and middle-income countries^1^ and present a risk to high-income countries.^2^ More than a billion people are infected each year by VBD resulting in more than a million deaths.^3^ Most VBD do not have an available vaccine or specific treatment, making vector control essential to preventing transmission.^3 4^

In order to be effective and sustainable, vector control programmes must educate and involve the community and adapt to diverse cultural and environmental contexts.^4^ Programmes that fail to do this encounter social and cultural norms that impede their execution.^5^ Historically vector control programmes have employed men and women in roles that follow traditional gender norms.^6–9^ Men, as compared with women, are more likely to be involved with vector control methods that employ physical labour such as reducing vector habitat, spraying insecticides and improving sanitation.^6 7^ In contrast, studies have demonstrated that women are more actively involved in organising and educating their local communities^6 8^ Relatively few studies have examined how local gender norms affect participation in workforce development programmes,^10^ particularly those aimed at reducing VBD. However, strictly adhering to binary gender roles for employment in vector control programmes could impede a vector control programme. For example, indoor residual spraying is conducted in all sleeping spaces in a dwelling. Cultural norms may not permit a woman to allow an unknown adult male to enter the house, let alone the private spaces within the house such as the bedroom.^11^ Including women on an indoor residual spraying team may address this concern. While inclusion of women may help address some programme barriers, it could raise others. Gender norms may prohibit women from staying overnight to undertake mosquito sampling, travelling alone, being supervised by males and working with insecticides.^12^ Additionally, women-centred vector control programming has led to community pressure to include males.^13–17^

There are two primary reasons for gender mainstreaming vector control programming: (1) providing equal access to work for both men and women as is recommended under the Sustainable Development Goals 5 and 8^18^ and (2) enhancing women’s participation, which may lead to improved vector control programme outcomes, as has been demonstrated in other development programmes.^19–28^

Increasing access for women to participate in economic opportunities and earn an income can influence their agency through economic independence.^28^ Further reaching impacts have also been noted. Some studies reported a shift in attitudes towards pro-gender equality leading to a reduction in violence against women.^24 26 27^ These reports indicated that a decrease in economic stress felt by the patriarch leads to a reduction in domestic violence. However, not all development programmes that sought to increase women’s participation and social status have encountered success. For example, some programmes that sought to increase women’s bargaining power and negotiation skills and decrease household poverty observed an increased risk of domestic violence towards women.^19 20 22 23 25^ In some cases, it was hypothesised that men were dissatisfied with the shifting household roles; therefore, they used violence to control income and household resources. However, one report observed community backlash towards men who were angered by the increased economic status of women.^21^

Enhancing women’s participation may also increase the effectiveness of the vector control programme. Anecdotal evidence suggests that when women are involved in the design, delivery and adoption of health interventions, there may be greater acceptance and compliance^9^ and, thus, greater impact. Other development efforts that have a longer history of gender mainstreaming, including water supply, sanitation and agriculture, have demonstrated significant benefits of women’s involvement.^29 30^ For example, in reforestation programmes that were women-led, regrowth was greater and attributed to better adherence with the regulations and women’s collaborative strategies.^31^ While men’s and women’s roles are distinctly different in forestry, their involvement has led to increased food security and the sustainability of the forest.^32^ Water development programmes have also demonstrated various benefits when women were involved, including more cost-effective solutions that improved access and reduced corruption.^33^

**Figure 1:**
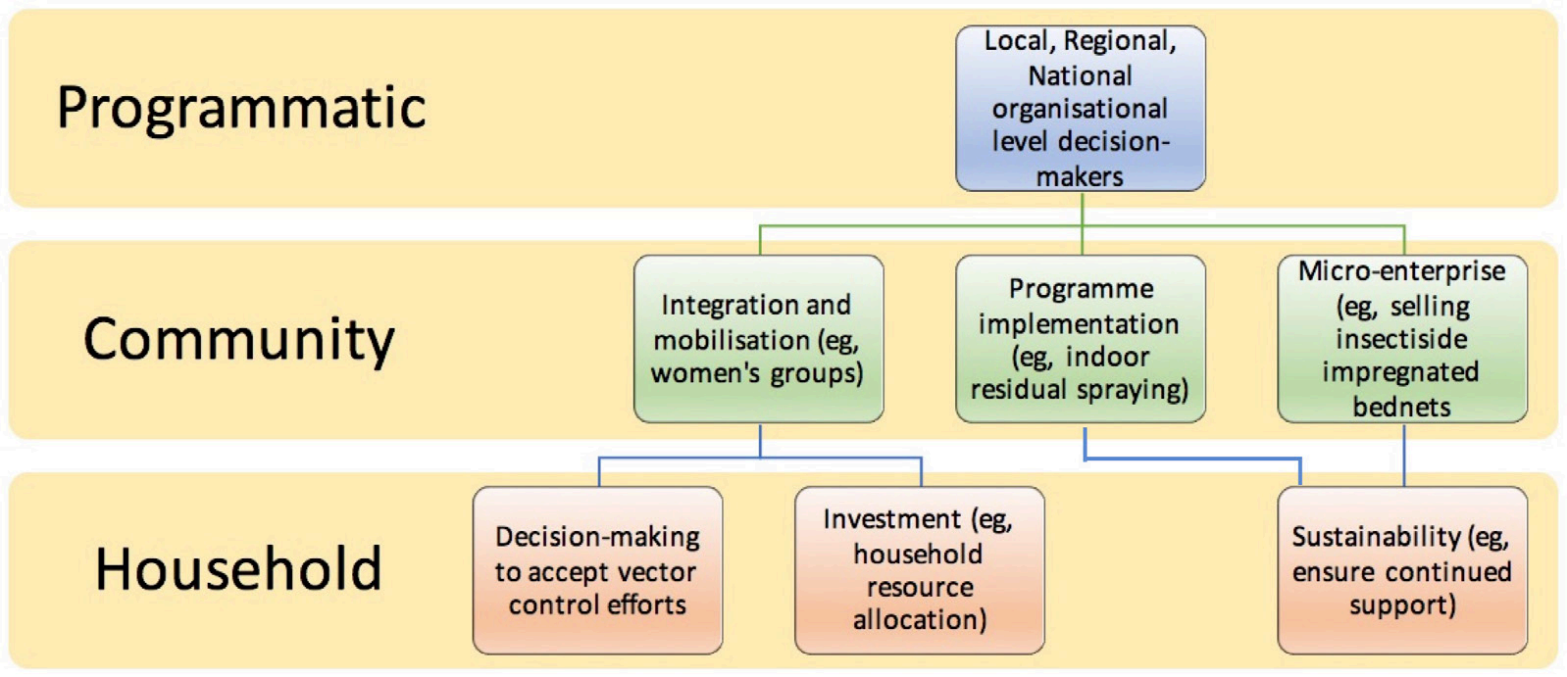
Overview of the integration of women into multiple levels of vector control.

Although the scientific literature has called attention to the important role women may play in vector control, no published literature review has assessed the scope of women’s engagement in vector control.^11^ We propose that women can and should be involved in multiple areas of vector control implementation and leadership (figure 1). In this systematic review, we focus on identifying the ways that women are involved in vector control at the community level. Three primary realms of participation were identified at the community level: women’s involvement in integration of vector control activities into a community and mobilisation; the implementation of top-down programmes such as indoor residual spray programmes and the distribution of long lasting insecticide treated bed nets and micro-enterprises to sell and distribute vector control products.

### Objective

The objective of this systematic review was to assess and critically summarise current vector control strategies that engaged women at the community level. In this instance, community-level programmes are defined as those that are implemented in the field at a unit broader than the individual woman’s household and narrower than national, state or province level interventions. The geography and scope of the community may vary.

## Methods and analyses

### Studies

Studies that were included were required to report involving women in vector control strategies such as indoor residual spraying, mass trapping, habitat modification, community-integrated vector management, larviciding and/or bed net distribution at the community level and higher. No study was excluded based on study type, quality, geographical location or publication status. Only studies published in English were included in this review. This study is reported according to the Preferred Reporting Items for Systematic Review and Meta-Analysis (see online supplementary appendix A).^34^

10.1136/bmjgh-2017-000366.supp1 Supplementary file 1

### Search methods for identification of studies

The following databases were searched from inception to 21 December 2015: Pubmed/Medline; Web of Science; EBSCOhost-Medline, PsycINFO, Gender Studies and Popline. The search criteria can be found in online supplementary appendix B. A broad range of keywords that focused on women, gender and vector control strategies were first established in PubMed/Medline and then adapted to each individual database to identify a variety of articles from different fields. Because not all organisations submit publications for peer-review, we also searched ‘grey literature’ by assessing websites that focus on international development and vector control (see table 1). Free text searches combining a variety of search terms such as *women AND ‘vector control’* or *female AND ‘vector control’* were conducted on each website (see full list in box). The first 100 titles, sorted by relevance, were assessed for inclusion. Because not all websites were able to be searched using this standard procedure, some websites were searched using Google’s advanced searching option. Websites were entered into the ‘site or domain’ line and the search terms entered into the ‘all these words’ line. Searches were sorted by relevance and the first 100 titles were screened for inclusion.

10.1136/bmjgh-2017-000366.supp2 Supplementary file 2

**Table 1:** Grey literature search

Organisation	Website
African Development Bank	http://www.afdb.org/en/
Asian Development Bank	http://www.adb.org
Australian Agency for International Development	http://www.dfat.gov.au/aid/
Bayer	http://www.bayer.com
CARE International	http://www.care-international.org
CARE US	http://www.care.org
CropLife International	http://www.croplife.org
Department for International Development	http://www.gov.uk/government/organisations/department-for-international-development
European Centre for Disease Prevention and Control	http://www.ecdc.europa.eu
Fiocruz (Brazil)	http://portal.fiocruz.br/en
Gates Foundation	http://www.gatesfoundation.org
Innovative Vector Control Consortium	http://www.ivcc.com
Inter-American Development Bank	http://www.iadp.org
International Initiative for Impact Evaluation	http://www.3iempact.org/
International Institute for Environment and Development	http://www.iied.org
International Livestock Research Institute	http://www.ilri.org
International Monetary Fund	http://www.imf.org
Oxfam	http://www.oxfam.org
President’s Malaria Initiative	http://www.pmi.gov
Roll Back Malaria	http://www.rollbackmalaria.org
The Global Fund	http://www.theglobalfund.org
United Nations Development Program	http://www.undp.org
United Nations	http://www.un.org/en/
United States Centers for Disease Control and Prevention	http://www.cdc.gov
United States Agency for International Development	http://www.usaid.gov
Vector Control Research Centre	http://www.vcrc.res.in
Vestergaard Frandsen	http://www.vestergaard.com
Wellcome Trust	http://www.wellcome.ac.uk
Women Organizing for Change in Agriculture and Natural Resource Management	http://www.wocan.org
World Agroforestry Centre	http://www.worldagroforestry.org
World Bank	http://www.worldbank.org
WHO	http://www.who.int
World Vision	http://www.worldvision.org

Box **Website search term**

- female AND “malaria control”
- female AND mosquito
- female AND “vector control”
- women AND “malaria control”
- women AND mosquito
- women AND “vector control”

### Selection of studies

Articles were included in this review if they discussed women’s participation in vector control. After duplicates from the initial search were removed, full abstracts that met the defined inclusion criteria were screened independently by two authors (JG and KB) using a predetermined inclusion form. A third reviewer (KE) was consulted when disagreement or uncertainty about an article’s inclusion occurred. Reasons for exclusion based on titles and abstract were documented. All articles that met the predetermined inclusion criteria were retained. Both backward and forward snowballing techniques were used to ensure that all relevant articles were identified.^4^ Backward snowballing is a technique where references in an article that met inclusion criteria were screened for additional relevant articles. For forward snowballing, which involved searching for studies that referenced the included study, Google Scholar was used. Forward and backward snowballing was also applied to relevant review articles. It is important to note that only articles that discussed engaging women directly in vector control, not infectious disease treatment or only VBD education, were retained.

### Data extraction and management

Multiple reviewers (JG, KC and AO) used a predetermined form for data extraction. A third reviewer (KE) was consulted if consensus could not be reached by at least two reviewers. When sources contained limited information, data were not reported.

### Assessment of risk of bias and study quality in included studies

Because all data sources were included in this systematic review (ie, peer-reviewed articles, non-peer-reviewed reports, websites and so on), no studies were excluded based on study quality or bias. Quality of articles published in a peer-reviewed journal was assessed using the GRADE approach.^35^ The GRADE approach assigns a quality level of high, moderate, low and very low with randomised control trials starting at high and observational studies starting at low. Websites were not assessed for quality.

## Results

### Peer-reviewed literature

**Figure 2:**
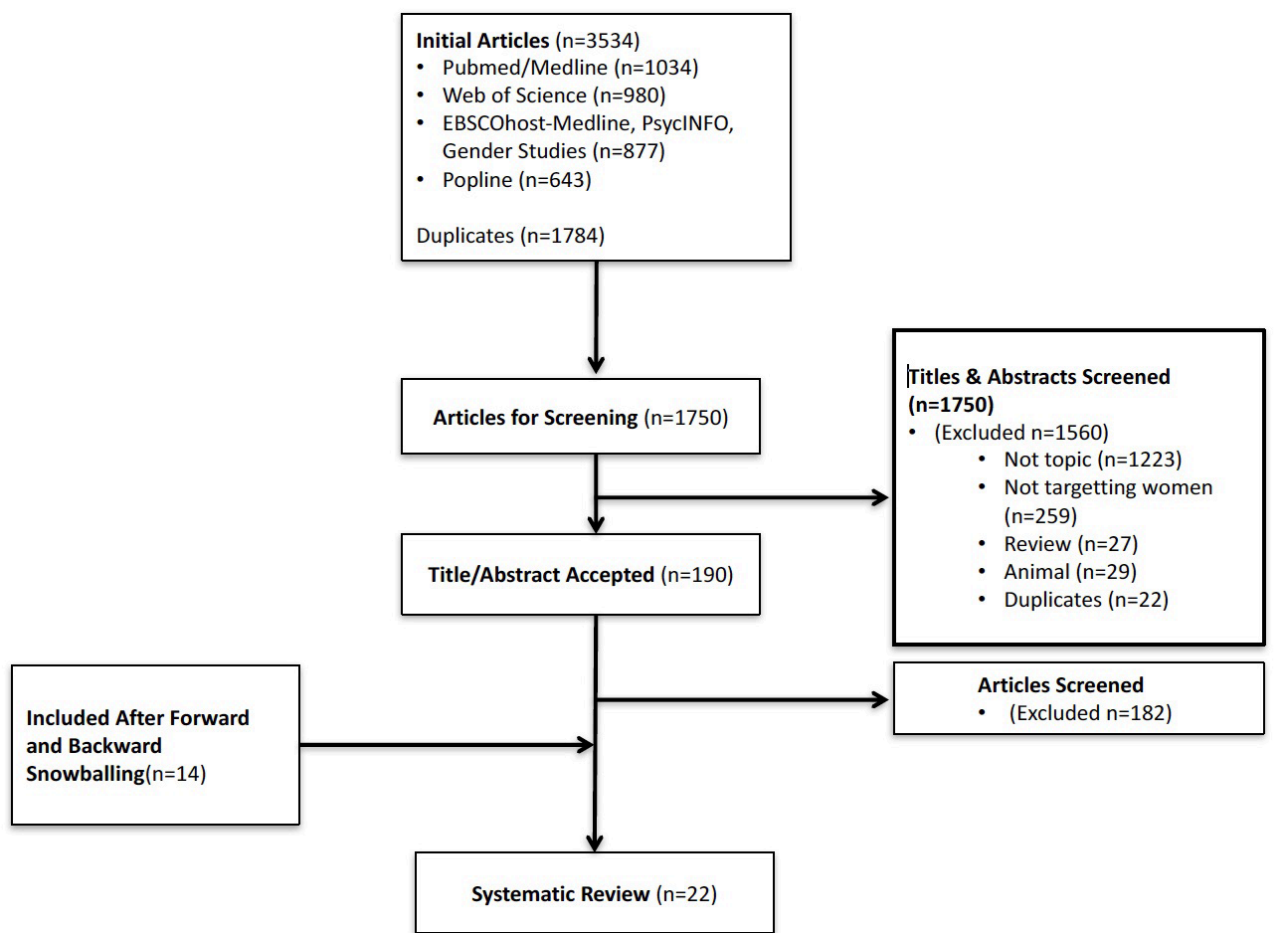
Literature search and study selection.

The initial literature search within the six databases yielded 3534 articles of which 1784 were duplicates (figure 2). Of the 1750 unique articles screened, 1560 were excluded based on their title and abstract and 181 were excluded after reviewing the study itself. A total of 23 articles^6 15–17 36–54^ representing 17 unique studies^6 15–17 37 38 40 42–44 46–51 53^ were included in this review. Nine studies were retained from the original search,^6 15 36 44–47 52 54^ and 14 were added from forward and backward snowballing.^16 17 37–43 48–51 53^ Ten studies were conducted in low-income countries as defined by the human development index,^6 15 37 40–45 50^ eight in medium-income countries,^16 36 38 39 46 48 53 54^ four in high-income countries^17 47 49 51^ and one in multiple countries of varying incomes.^52^ Three studies discussed involving women in vector control programmes for human African trypanosomiasis (HAT),^42 43 50^ 8 discussed dengue,^16 36 39 46 47 52–54^ 10 malaria,^15 17 37 38 40 41 44 45 48 49^ 1 schistosomiasis^51^ and 1 water-borne diseases including both malaria and schistosomiasis.^6^ Study quality varied greatly, with six articles showing high study quality,^36 38 39 48 52 54^ 13 moderate,^6 15–17 41–45 47 49 50 53^ 3 low^40 46 51^ and 1 very low.^37^ Results across studies could not be compared due to inherent differences in their design, interventions and outcomes.

### Website searches and personal communications

Thirty-three websites were searched in order to assess current strategies of engaging women in vector control. Five unique programmes were discovered during this grey literature search and included in this review.^14 55–58^ Two additional programmes were added based on personal communications.^13 59^ Two programmes were conducted in low-income countries,^13 56^ one each in a lower middle income country,^14^ a medium-income country^58^ and an upper middle income country^57^ and two in multiple countries of varying incomes.^55 59^ Three studies discussed involving women in vector control programmes for malaria,^55 57 59^ and four discussed including women in general vector control of mosquitoes.^13 14 56 58^ Due to the inconsistent reporting of information, study quality was not assessed on the programmes discovered during this search of websites and personal communications.

### Women’s engagement, categorisation of themes

Studies were categorised into the three main themes identified a priori (figure 1) and further divided by geographic area. Overviews of the studies identified in each theme are provided below (see online supplementary file for details on articles from the database search).

10.1136/bmjgh-2017-000366.supp3 Supplementary file 3

### Micro-enterprise programmes in vector control

Six programmes were identified that engaged women in micro-enterprise to create and/or provide sustainable delivery of vector control products.^14 15 17 40 41 59^ Four of them were conducted in sub-Saharan Africa,^15 40 41 59^ one in Latin America^17^ and one in Asia.^14^ All of the programmes were focused on malaria control. Five of the six were engaging women and in some cases men and women,^17 40^ in either making, selling or treating insecticide-treated bed nets.^14 15 17 40 41^ Of those, the Tupendane study in Tanzania also provided women with polystyrene beads to sell that acted as a layer to prevent mosquito development in latrines and septic tanks.^15^ These differed slightly from the Mama Mosquito project being carried out in Senegal where women, generally over age 40, were trained on the proper procedures and safety protocols to administer indoor residual spray.^59^ Several of the studies discussed the success of the programmes in terms of women being the primary holders of leadership positions in community-based cooperatives^17^ and the ability of the programmes to provide affordable products and services for the communities in which they were engaged.^17 40 59^ None of the studies provided head to head comparison with similar male-led programmes and long-term follow-up had not been undertaken.

### Community-engaged vector control strategies

Of the programmes identified, 18 were targetting smaller geographic areas and employing women in one or more of the following: (1) education, outreach and community building to reduce vector habitat and vector-human contact; (2) habitat identification and destruction and (3) reduction in vector density through trapping, treatment or other means.

#### Education, outreach and community-building

The majority (n=10) of community-based programmes that involved women were developed to train volunteers about vector control who subsequently disseminated this knowledge to others.^6 16 36 38 44 45 47–49 54^ Some of these also had a broader component in which organisational structures were developed to oversee and monitor the community-level vector control activities.^16 44 48 49^

In sub-Saharan Africa, three programmes were identified that involved women in outreach and education. None of these programmes solely targeted women’s involvement, but women were specifically identified as being involved. These included a project in Kenya that compared vector indices after implementation of integrated vector management in two areas, Malindi and Nyabondo. In Malindi, multiple stakeholders were involved including schools, hospitals, women’s groups and malaria scouts (mostly women) who helped educate the public. They attributed the relatively higher decline in malaria cases to increased awareness and reduction in breeding sites. The programme did not specifically compare success by gender, but women were highly involved in the evidence-based decision making, advocacy and social mobilisation, vertical and horizontal collaborations, integrated approaches and capacity building that contributed to the success of the programme.^41 45^ In neighbouring Sudan, the Blue Nile Health Project (BNHP) carried out between 1980 and 1990 engaged women to reduce water-associated illnesses including schistosomiasis and malaria by acting as (1) health educators; (2) village health committees and (3) recipients of the BNHP. Women constituted 75% of the health educators at the BNHP, 40% of the village health committees and 40% of recipients. Study results indicated that health educators and village health committees played a key role in the motivation, organisation and health education of local campaigns prior to vector control implementation. Compared with their male counterparts, female employees of the BNHP played a more active role in health education programmes, self-protection campaigns and treatment of VBDs.^6^ In Tanzania, community participation in an insecticide-treated bed net programme involved the development of effective messages that were communicated to community members by posters, skits, talks and lessons. This resulted in a high level of participation, and more people were willing to purchase bed nets and insecticides. A system for accumulation of capital was developed within each village, and a management structure was set up and defined in a locally developed constitution. However, women were only nominated by the community to lead vector control activities when the village was specifically asked to nominate a woman.

In South and Southeastern Asia, seven programmes were identified in which women were involved in community outreach and organisation of vector control.^16 39 41 50–52 56^ Most were engaged in dissemination of educational messaging through multiple channels to the broader community.^39 41 51 52^ However, two of the programmes, one in Indonesia^56^ and another in Thailand,^50^ were focused on empowering women to become family leaders and decision-makers about vector control within their own household and among family members. One of the programmes developed a stronger structure in which Accredited Social Health Activists (ASHA), females from local communities with little formal education and no previous experience were supervised by community health workers to deliver messages on the use of bed nets. Additional women from self-help groups measured bed net use in the community. Outcomes were improved in this hierarchical structure as compared with intervention arms with only the ASHA.^41^ Several of the programmes went beyond education-only strategies to address vector control and included community clean-ups,^39^ breeding source management^51^ and specific training to identify the malaria vector.^52^ Several of the programmes had demonstrable results including improved knowledge about dengue,^39^ higher bed net use, lower malaria parasite densities,^51^ reduction in *Ae. aegypti* indices.^50^ However, in Cambodia, qualitative assessments of women’s willingness to engage in community-based vector control indicated that there had been a shift in attitude towards working beyond their own households without compensation. They attributed this in part to ongoing political unrest. Women reported that community-based dengue control occurred, but, in practice, people had limited resources to participate in these activities.^15^

#### Vector habitat identification and reduction

Four studies were identified in which women were involved in the identification and elimination of vector habitat.^40 42 49 55^ One was in sub-Saharan Africa,^40^ two were set in southeast Asia^42 55^ and one was in Latin America,^49^ In Tanzania, women made up nearly half (44%, n=28) of community-owned resource persons who were assessed for their ability to detect mosquito breeding sites. Resource persons living in or near their areas of work found a higher proportion of wet habitats containing *Anopheline* and *Culicine* larvae than those that lived outside their areas of responsibility. Both studies in southeast Asia focused on the identification of *Ae. aegypti* habitat. Women were exclusively engaged in Indonesia,^55^ and both men and women were engaged in the Philippines.^42^ In the all-female programme,^55^ Breteau indices were reduced significantly after 6 months. No reports of the outcomes were made in the Philippines studies but there was one all woman team involved in the programme that reported a sense of personal fulfilment from the training they received and the household visits performed as part of the study. In Nicaragua, community health workers, 90% of which were women, were taught how to identify, count, record and destroy foci within the community. Female participants indicated that women were better equipped to perform the role of vector control within the community as they had better social and negotiation skills and empathy than men. Women also indicated they had more intimate knowledge of the household.^49^

#### Reduction in vector density through trapping

Three studies were identified that used trapping strategies to reduce vector densities, and all were targetting control of tsetse flies that carry HAT. All were focused in eastern Africa including Uganda,^45^ South Sudan^46^ and Tanzania.^53^ They ranged in their level of women’s involvement. The programme in western Tanzania involved women the least with participants in HAT control; males constituted 92.9% of participants. Reasons for the disparity included reports that men were perceived to be stronger (40%) and earned more money than women, but were more likely to suffer from the disease. In South Sudan, women were directly recruited including traditional birth attendants and women’s groups. They were involved in screening, treating and setting up traps in high vector-density areas.^46^ The average number of flies caught in each trap dropped significantly, from 25 to 3. The prevalence of trypanosomiasis was reduced from 9% to 2%. The success of the programme was attributed to creating traps that were easily constructed from local materials, easy to set up and easy to maintain. In Uganda, local women were recruited to carry out HAT control by placing ‘tiny targets’ along river banks (Village A). These activities were compared with another village where experts and field technicians ran the control programme (Village B). Overall, women were willing, motivated and organised to successfully manage the tiny targets operation. Their sense of ownership of the project and sense of empowerment increased, which proved to be cost effective. Participants in Village A perceived the intervention as feasible. Because communities maintained them, more tiny targets were functional at 6 months postdeployment in Village A than in Village B. At the end of the intervention, women requested replacement targets to deploy the following year. No quantitative comparison has yet been published.^45^

#### Reduction in human-vector contact

One study was identified in which women were engaged in producing mosquito repellents from local plants. In Mozambique, women’s organisations created Climate Change Health Kits for traditional healers and healthcare workers to provide indigenous and essential plants to ward off mosquitoes and treat medical ailments.^56^ No other information on the role of women or outcomes of this programme was found.

#### Highly integrated strategies

While many of the programmes had multiple targets, one study in particular focused on a wide range of interventions as well as a diverse set of countries.^59^ This programme was focused on bio-social-ecological control of dengue in India, Sri Lanka, Indonesia, Myanmar, Philippines and Thailand.^59^ The common goals across the study sites were to^1^: design, conduct and evaluate multipartnership interventions with emphasis on community involvement and to^2^ assess the effect of the intervention on partners and the vector populations. By developing community-based interventions aimed at reducing dengue vector breeding and viral transmission, the study site mobilised and empowered women’s groups as well as student and community groups to decrease vector density. Strong involvement of women in the intervention was reported in all but the Philippines. Although the article reports that women actively participated in the project, authors also acknowledge that women played a minor role in decision-making. Only information regarding women’s involvement in India was included in this article. In India, women were instrumental in delivering non-insecticidal water container covers for cement tanks, drums and barrels. Overall, peoples’ knowledge of dengue transmitting mosquitoes was associated with reduced mosquito breeding and production, attributed to increased self-protection with domestic insecticides. Vector control measures substantially reduced the larval/pupal indices and ‘pushed’ mosquito breeding to alternative containers. The programmes led to the formation of community groups and other public and private partners.

### Top-down implementation of national control programme strategies

Women were also involved as employees of or volunteers in top-down national or subnational level programmes. Two of the five identified programmes were employment opportunities^13 58^ and three were volunteer.^51 57 58^ Both programmes that involved employment were cross-country initiatives for malaria control employed in sub-Saharan Africa; VectorWorks and the African Indoor Residual Spray programme (AIRS). Both began with a systematic analysis of gender norms and women’s involvement in the prevention and control of malaria and both specifically targeted increasing the involvement of women. Subsequent strategies were developed to enhance their role. Gender focal points were identified to lead country-level efforts and inclusive policies were enacted. The VectorWorks programme is involved in bed net distribution and strives for equal participation of men and women. Specific policies were not available for review.^13^ The AIRS programme policies were aimed to attract and retain female employees by adapting the physical work environment to ensure privacy for women (ie, safe changing areas, bathrooms and showers for women to use); implementing policies aimed at creating a hospitable work environment; ensuring safety and job security during pregnancy and encouraging women to take supervisory positions.^55^ Both programmes are consistently collecting data to track progress and identify areas of improvement. Neither has reported quantitative results of their efforts.

In addition to paid positions, women have also been recruited as part of national-level volunteer efforts to control VBDs.^51 57 58^ The longest standing of these was the Guangxi Schistosomiasis Control programme run by the Chinese government.^51^ While women were not the sole target for involvement, the Women’s Federation made the project a priority on their local agenda and were involved in activities such as scraping earth, piling up compost, filling up gullies, transforming low-lying land, burning materials in which snails breed, tunnelling through mountains to drain waterlogged lands and reclaiming wasteland. In Iran and India, community health workers and volunteers were recruited for national vector control programmes.^58^ In Iran, most volunteers were women, and the programme in India only included female volunteers. In both programmes, volunteers were primarily trained as health educators, while also receiving specific training on recognising vector habitat and generating and coordinating solutions with residents and community members.

## Discussion

This systematic review of scientific and grey literature demonstrated that women are successful at participating in and leading community-level vector control programmes. Although the systematic collection of data on the effectiveness of engaging women in vector control is currently inadequate to draw definite conclusions, most programmes qualitatively supported that programmes benefited from increasing women’s participation in vector control. Evidence suggested that using women in vector control strategies can create economic development opportunities for women as well as provide access to communities that may otherwise be hard to reach. Often, a programme’s success was contingent on working within existing networks or finding women in the community who were considered trustworthy.^16 40 43^ However, difficulties did occur while attempting to engage women in vector control as indicated below.

### Common difficulties encountered

Many studies discussed multifaceted difficulties that cannot be overlooked. Common barriers to women’s participation in vector control included some that were specific to women: (1) the need for flexibility in the workplace as cultural norms often meant women needed to attend to domestic activities before participating in economic activities^6 15 36 37 39 42 44 45 47 52 54^ and others that were broader challenges to vector control; (2) the economic burden of starting a business^41 44 45^; (3) the difficulties of creating a self-sustaining business^15 43 44 47 48^; (4) the political and legal difficulties that were often outside the control of the researchers, the women participating in vector control and the local community leaders^13–17^; (5) the lack of equipment and supplies^37 44^ and (6) the need for general financial and managerial training.^15 41 44 45 50^

### Recommendations

Despite the difficulties encountered, studies provided specific recommendations for increasing women’s participation in vector control. The first recommendation was to understand the local relationship between traditional and current gender norms while promoting flexibility and economic incentives.^6 60^ This could help determine how best to continue to help women implement vector control in communities and households.^6 60^ Several studies that used women as volunteers indicated that women often had domestic activities or work commitments that decreased the time they had available for volunteering.^6 15 36 37 39 42 44 45 47 52 54^ Finding ways to promote flexibility so women can balance household commitments and obtain employment is essential to increasing women’s ability to participate. Furthermore, creating economic incentives, instead of relying solely on volunteer work, may increase women’s participation and increase gender equity.

The second recommendation centred around the economic burden of starting a business. Multiple projects found success by donating the initial supplies needed for starting a small vector control business.^15 17 40^ Women were then able to sell the initial products for a profit and buy supplies to replenish their inventory. This business model allowed women to build a self-sustaining business without the need for continued government or foreign aid. Additionally, because some studies indicated theft of either money or supplies,^15 17 53^ programmes may need to find innovative solutions to keeping finances and supplies safe.

The third recommendation was to create clear leadership and direction while trying to implement a vector control programme.^36 38 39 52 54^ As Das and colleagues indicated, community-based interventions that provide the supportive supervision of community health workers with community mobilisation can lead to an increase in efficacy, acceptability and utilisation of community health volunteers.^38^ Because effective leadership and management is essential to the success of any programme, programmes that include training on management and leadership skills may be essential for long-term success.

Last, the fourth recommendation was to build a partnership between the villagers, district authorities and the central government. Because women are often vulnerable to policies and norms that are not supportive of women-centred businesses, many studies discussed the difficulty of creating activities that included women, while also dealing with political pressure in the communities to include men.^13–17^ Furthermore, programmes often encountered difficulties based on national policies that impaired programmes’ sustainability such as high import taxes on insecticide.^13 16 17 36 40 41 45 51 52^ By establishing multisectoral collaboration between all parties, programmes may be able to leverage resources and implement policy changes to help protect the rights of women while aiding their ability to keep programmes sustainable.

### Strengths and limitations

This is the first systematic review to assess the evidence for engaging women in vector control. To provide a comprehensive overview of the activities published, we included literature published in peer-review journals and grey literature. This study, however, is not without its limitations. The authors chose a variety of websites that focus on international development strategies. The list of websites is not exhaustive; however, it does provide an overview of current projects that are using women for vector control. Including grey literature allows us to observe the importance of improving collaborations between academic institutions and other organisations, as both have established that including women in vector control should be made a priority. They have also both demonstrated that women are often conscious of their community needs and capacity which makes them successful when given the resources and opportunities to participate in vector control strategies. A limitation of this review is that using women to combat malaria,^61–66^ dengue^67^ and schistosomiasis^68^ was mentioned in a variety of articles; however, no detailed information could be extracted. Articles in both peer-reviewed journals and grey literature often lacked detailed information of the roles women are undertaking in vector control programmes.

Another key limitation is the generalisability of the results from specific programmes to other sociocultural contexts. Global reports demonstrate that there is significant geographic variability in gender norms and the status of women. For example, equal inheritance rights for sons and daughters vary greatly by WHO region, ranging from no countries in the Middle East and North Africa (MENA), 33% of countries in South Asia, 76% in sub-Saharan Africa, 85% in East Asia, to 100% of countries in Latin America and the Caribbean (LAC).^33^ Further, support infrastructure for women in the workforce who have children varies with maternity leave funded by social programmes in 24% of MENA countries to 62% in LAC.

More studies and programme evaluations are needed that are specifically designed to analyse magnitude of change in the outcomes when women are included in vector control.

### Implications for policy

VBD control requires an integrated vector management strategy. Women are more likely to stay and live within rural communities and are often differentially impacted by some VBDs including malaria, schistosomiasis and trypanosomiasis. The employment of women, particularly grandmothers, has been beneficial to expansion of development activities, including solar energy.^69^ Policies to train individuals who reside in and are likely to stay within a community should be investigated. Empowering women often begins with income-generating activities that give women more economic power within society.^9^ Increasing policies and funding that engage women in vector control could provide an economic shift for communities and decrease poverty in households where women are able to establish an income. Policies that create economic opportunities for women to participate in and develop businesses are essential. Equally important are policies that focus on the sustainability of vector control business development.

## Conclusion

This systematic review demonstrated that women can be successfully engaged in vector control programmes at the community level in both income generating vector control activities, including micro-enterprise, paid community-level and state and national-level programmes and volunteer positions. There is a significant evidence gap for the relative effectiveness of vector control in women-centred vector control programmes. Rigorous comparative effectiveness studies need to be conducted.
