# Supplementary material for: Current strategies and successes in engaging women in vector control: a systematic review
Source: BMJ Glob Health. 2018 Jan 7;3(1):e000366. doi: 10.1136/bmjgh-2017-000366 (PMC5838394; doi:10.1136/bmjgh-2017-000366)
Supplement: Supplementary file 2 [file bmjgh-2017-000366supp002.pdf]

APPENDIX B: Search strategy

The following databases will be searched from inception to the latest issue: Pubmed/Medline; Web of Science; EBSCOhost-Medline, PsycINFO, Gender Studies; and Popline.

All searches will be adapted from PubMed/Medline.

1. "gender"[tiab] OR "gender roles"[tiab] OR "gender role"[tiab] OR "gender identity"[Mesh] OR "women"[Mesh] OR “women”[tiab] OR "women's groups"[tiab] OR "Women/utilization"[Mesh] OR “empower”[tiab] OR “empowerment”[tiab] OR “women, working”[Mesh] OR “working women”[tiab]

2. (("Source reduction"[tiab] OR "larval control"[tiab] OR "tick control"[tiab] OR "fly control"[tiab] OR "pest control"[tiab] OR "pest management"[tiab] OR "trash disposal"[tiab] OR "ovitraps"[tiab] OR "Entomopathogenic fungus"[tiab] OR "BG sentinel traps"[tiab] OR "insecticide spraying"[tiab] OR "biological control"[tiab] OR "chemical control"[tiab] OR "larvivorous fish"[tiab] OR "Wolbachia"[tiab] OR "vector management"[tiab] OR "integrated vector management"[tiab] OR "indoor residual spray"[tiab] OR "IRS"[tiab] OR "Insecticide-Treated Bednets/utilization"[Mesh] OR "copepods"[tiab] OR "Bacillus thuringiensis israelensis"[tiab] OR "Bti"[tiab] OR "Spinosad"[tiab] OR "Lysinibacillus sphaericus"[tiab] OR "pyrethroids"[tiab] OR "attractive toxic sugar bait"[tiab] OR "sterile insect technique"[tiab] OR "insecticide-treated materials"[tiab] OR "insecticide treated materials"[tiab] OR "Culicidae"[Mesh] OR "Mosquito Nets/utilization"[Mesh] OR "mosquito control/education"[Mesh] OR "mosquito net"[tiab] OR "mosquito nets"[tiab] OR "curtains"[tiab] OR "spatial repellent"[tiab] OR "LLIN"[tiab] OR "long lasting insecticidal nets"[All Fields] OR "insecticide treated net"[tiab] OR "ITN"[tiab] OR "larvicide"[tiab] OR "arthropod borne"[tiab] OR "vector control"[All Fields] OR "Insect Vectors"[Mesh] OR "vector control management"[All Fields] OR "vector control strategies"[All Fields] OR "environmental management"[tiab] OR "fogging"[tiab] OR "fogger"[tiab])

3. 1 AND 2
