# Supplementary material for: Current strategies and successes in engaging women in vector control: a systematic review
Source: BMJ Glob Health. 2018 Jan 7;3(1):e000366. doi: 10.1136/bmjgh-2017-000366 (PMC5838394; doi:10.1136/bmjgh-2017-000366)
Supplement: Supplementary file 3 [file bmjgh-2017-000366supp003.pdf]

Supplement: Description of articles from database search

Arahman S, Mohamedani AA, Mirgani EM, Ibrahim AM. Gender aspects and women's participation in the control and management of malaria in central Sudan. *Soc Sci Med*. 1996;42(10):1433-1446.

**Location of Study**	Central Sudan
**Human Development Index**	Low
**Vector**	Multiple
**Disease**	Multiple diseases that are associated with water (including malaria)
**Study Design**	Cross sectional
**Funding Agencies**	Sudan Government; World Health Organization
**Ethics Approval**	None Reported
**Study Quality**	Moderate
**Study Description**	The Blue Nile Health Project (BNHP) was launched between 1980-1990 to decrease water associated disease such as malaria and schistosomiases in Central Sudan. In order to understand the impact of women's roles within the BNHP, this study aimed to describe the gender related aspects that defined women's role and participation in the project. Cross-sectional surveys were given to women who performed three roles in the project 1) health educators; 2) Village health committees; 3) recipients of the BNHP. Women constituted: 75% of the health educators at the BNHP; 40% of the village health committees; 40% of recipients of the BNHP. Study results indicated that health educators and village health committees played a key role in the motivation, organization and health education of local campaigns prior to vector control implementation. Compared to their male counterparts, women employees of the BNHP played a more active role in health education programs, self-protection campaigns, and treatment of vector borne diseases. Women reported that household commitments and difficulty communicating with the public were the two major difficulties they encountered while working with the project. Arahman and colleagues recommend that future studies attempt to persuade women to be involved in health program activities.
**Difficulties encountered**	Women encountered problems with performing the work because of household obligations as well as issues with communication in a public setting.
**Study Recommendations**	There is a need in the community for a higher level of health education. Moreover, health workers should encourage women to become more involved in health programs.

Supplement: Description of articles from database search

Arunachalam N, Tyagi BK, Samuel M, Krishnamoorthi R, Manavalan R, Tewari SC, et al. Community-based control of *Aedes aegypti* by adoption of eco-health methods in Chennai City, India. *Pathog Glob Health*. 2012;106(8):488-496.*

**Location of Study**	Chennai City, India
**Human Development Index**	Medium
**Vector**	*Aedes aegypti*
**Disease**	Dengue
**Study Design**	Randomized Cluster Trial
**Funding Agencies**	International Development Research Centre of Canada
**Ethics Approval**	Ethics Committee of the Centre for Research in Medical Entomology; Ethical Review Committee of the World Health Organization
**Study Quality**	High
**Study Description**	A study was carried out in Chennai City, India to investigate the efficacy, favoring, and limiting factors of a community-based dengue vector control intervention. The main outcome being considered was a reduction in pupal indices. The study design was a randomized control trial of 10 clusters of 100 homes each matched with 10 clusters of a control arm. The intervention arm received a community-based environmental treatment approach in which the following activities were carried out: clean-up campaigns, dissemination of information to school children, and community actors. Results of the study show that there was a recognizable increase in knowledge about Dengue in the community as well as a significant reduction in pupal indices. Women's self-help groups were utilized as part of community involvement in the intervention.
**Difficulties encountered**	Women showed a general view that they can't be involved in community organizations because of work at home as well as being very shy when talking with unknown males.
**Study Recommendations**	Community members should be involved (by targeted multiple stakeholders) in interventions in order to control dengue vectors.

* Part of the same research program as Espino F, Marco J, Salazar NP, Salazar F, Mendoza Y, Velazco A. Community-based dengue vector control: experiences in behavior change in Metropolitan Manila, Philippines. *Pathog Glob Health*. 2012;106(8):455-460; Sommerfeld J, Kroeger A. Eco-bio-social research on dengue in Asia: a multicountry study on ecosystem and community-based approaches for the control of dengue vectors in urban and peri-urban Asia. *Pathog Glob Health*. 2012;106(8):428-435; Tana S, Umniyati S, Petzold M, Kroeger A, Sommerfeld J. Building and analyzing an innovative community-centered dengue-ecosystem management intervention in Yogyakarta, Indonesia. *Pathog Glob Health*. 2012;106(8):469-478.

Supplement: Description of articles from database search

Chaki PP, Dongus S, Fillinger U, Kelly A, Killeen GF. Community-owned resource persons for malaria vector control: enabling factors and challenges in an operational programme in Dar es Salaam, United Republic of Tanzania. *Hum Resour Health*. 2011;9(1):1.

**Location of Study**	Dar es Salaam, Tanzania
**Human Development Index**	Low
**Vector**	*Anopheles* mosquito
**Disease**	Malaria
**Study Design**	Cross-sectional survey; mixed methods (observational)
**Funding Agencies**	Bill & Melinda Gates Foundation, Malaria Transmission Consortium; Wellcome Trust, Research Career Development Fellowship
**Ethics Approval**	None Reported
**Study Quality**	Very Low
**Study Description**	Community-owned resource persons (CORP) were followed by investigators in order to assess their ability to detect mosquito breeding sites. Information on demographics, reasons for participating, and general work performance was also collected about the CORP. Women made up 44% (n=28) of the 64 CORP. One finding of the study was that the proportion of wet habitats found by the investigator that had been reported by CORP was higher among CORP appointed by the government as opposed to those recruited by program administrative staff. Additionally, CORP living in/near their areas of work found a higher proportion of wet habitats containing Anopheline and Culicine larvae than CORP that lived outside their areas of responsibility. CORP were underpaid for taxing work. The members were given a difficult time to gain access to fenced compounds to ascertain mosquito breeding sites. This study recommends that fewer better-paid workers should be put in place to perform the duties of CORP workers. These employees should be appointed by the government and should come from a close proximity to where the work in being carried out. Information regarding any gender differences in the ability of CORP were not assessed.
**Difficulties encountered**	CORP were underpaid for taxing work. The members were given a difficult time to gain access to fenced compounds to ascertain mosquito breeding sites.
**Study Recommendations**	This study recommends that fewer better-paid workers should be put in place to perform the duties of CORP workers. These employees should be appointed by the government and should come from a close proximity to where the work in being carried out.

Charlwood D. Women's Groups and the Marketing of Health Interventions-a Tanzanian Experience. *Papua New Guinea Med*. 2008;51(3/4):102.

**Location of Study**	Tanzania
**Human Development Index**	Low
**Vector**	Mosquito
**Disease**	Malaria
**Study Design**	Observational
**Funding Agencies**	None reported
**Ethics Approval**	None reported
**Study Quality**	Moderate
**Study Description**	In 'Tupendane' women sold expanded polystyrene beads to use as a layer over the water in septic tanks and pit latrines. A blanket of beads a few centimeters thick prevents gravid females from laying their eggs and drowns larvae while allowing solid matter to pass through. They refloat in latrines that have temporarily gone dry and so do not need to be applied more than once. In 'Tupendane too' women impregnated and sold mosquito nets.
**Difficulties encountered**	The insecticide was blamed for a problem with low-quality nets and the money from the project was stolen.
**Study Recommendations**	None

Supplement: Description of articles from database search

Das A, Friedman J, Kandpal E, Ramana GN, Gupta RKD, Pradhan MM, et al. Strengthening malaria service delivery through supportive supervision and community mobilization in an endemic Indian setting: an evaluation of nested delivery models. *Malaria J*. 2014;13(1):1.

**Location of Study**	Odisha, India
**Human Development Index**	Medium
**Vector**	*Anopheles* mosquito
**Disease**	Malaria
**Study Design**	Randomized Control Trial
**Funding Agencies**	Spanish Impact Evaluation Fund, Department of International Development
**Ethics Approval**	Independent ethical committee in Bhubaneswar, India, per the guidelines of the Indian Council of Medical Research
**Study Quality**	High
**Study Description**	In Odisha, India, community mobilization teams and women's self-help groups were assigned to examine the effectiveness of community mobilization efforts in the promotion of malaria preventive behaviors. These behaviors included the usage of bednets as well as care-seeking by a designated and appropriate community health worker for febrile illnesses. The intervention was carried out in a total of 120 villages from two malaria-endemic districts, randomly assigned to three separate treatment arms. These treatment arms included a control, a community mobilization treatment, and a combination of a community mobilization treatment and supportive supervision. Community mobilization teams consisted of Accredited Social Health Activists: females from the local communities with little formal education and no previous experience before appointment. In the supportive supervision treatment arm, these health activists were supported with a goal of increasing capacity and providing resources. Moreover, the women's self-help groups played a role in the community mobilization treatment (both with and without supportive supervision) by measuring bed net use. Overall, significant improvements were found in the reported use of bednets in both treatment arms (versus the control) and in the treatment-seeking behaviors for febrile illnesses. Additionally, women from the combined interventions villages were more likely to have received timely and appropriate treatment for such illnesses than those in the community mobilization arm. These results suggested that a community-based intervention which combines both the supportive supervision of community health workers with community mobilization can lead to an increase in the efficacy, acceptability, and utilization of community health volunteers.
**Difficulties encountered**	None reported
**Study Recommen-dations**	A community-based intervention which combines both the supportive supervision of community health workers with community mobilization can lead to an increase in the efficacy, acceptability, and utilization of community health volunteers.

Supplement: Description of articles from database search

Espino F, Marco J, Salazar NP, Salazar F, Mendoza Y, Velazco A. Community-based dengue vector control: experiences in behavior change in Metropolitan Manila, Philippines. Pathog Glob Health. 2012;106(8):455-460.*

**Location of Study**	Masagana City, Manila, Philippines
**Human Development Index**	Medium
**Vector**	*Aedes aegypti*
**Disease**	Dengue
**Study Design**	Cohort
**Funding Agencies**	United Nations Children's Fund; United Nations Development Programme; World Bank; World Health Organization’s Special Programme for Research and Training in Tropical Diseases through a grant contribution from Canada’s International Development Research Centre
**Ethics Approval**	Institutional Review Board of the Research Institute for Tropical Medicine, Department of Health, Philippines; Ethics Committee of United Nations Children's Fund; United Nations Development Programme; World Bank; World Health Organization’s Special Programme for Research and Training in Tropical Diseases
**Study Quality**	High
**Study Description**	A study conducted in Masagana City, Philippines to observe community responses to the initiation of water container management for vector control of dengue in 84 households in a gated community (Village A) and 86 in an informal settlers’ community (Village B). The intervention was carried out by a study team, dengue control personnel and local health workers. Of these workers, one of the community’s task forces consisted of all women. As a result of the intervention, the female workers reported a sense of personal fulfillment from the training received and the household visits performed as part of the study.
**Difficulties encountered**	Village A had poor attendance and low collaboration. Problems in Village A included: gated communities, unknown addresses, and households not allowing visits. No household from Village A accepted the invitation to discuss the intervention. There was a general uncertainty of which sector was in charge of dengue control due to an absence of leadership.
**Study Recommendations**	In a country such as Cambodia, health education materials and methods need to be feasible and realistic when taking into account a community’s condition. The local perspective should be considered and integrated into vector-borne disease control.

* Part of the same research program as Arunachalam N, Tyagi BK, Samuel M, Krishnamoorthi R, Manavalan R, Tewari SC, et al. Community-based control of *Aedes aegypti* by adoption of eco-health methods in Chennai City, India. *Pathog Glob Health*. 2012;106(8):488-496; Sommerfeld J, Kroeger A. Eco-bio-social research on dengue in Asia: a multicountry study on ecosystem and community-based approaches for the control of dengue vectors in urban and peri-urban Asia. *Pathog Glob Health*. 2012;106(8):428-435; Tana S, Umniyati S, Petzold M, Kroeger A, Sommerfeld J. Building and analyzing an innovative community-centered dengue-ecosystem management intervention in Yogyakarta, Indonesia. *Pathog Glob Health*. 2012;106(8):469-478.

Fraser-Hurt N, Lyimo E. Insecticide-treated nets and treatment service: a trial using public and private sector channels in rural United Republic of Tanzania. *B World Health Organ*. 1998;76(6):607.

**Location of Study**	Ifakara, Kilombero District, United Republic of Tanzania
**Human Development Index**	Low
**Vector**	*Anopheles* mosquito
**Disease**	Malaria
**Study Design**	Cohort
**Funding Agencies**	Rotary Switzerland District 1980
**Ethics Approval**	Rotary Club Switzerland District 1980; Mr W. Vogtli
**Study Quality**	Low
**Study Description**	The Rotary Net Initiative, explored different sales channels for the distribution of insecticide-treated nets and the insecticide treatment service in a rural area with high malaria transmission. Several types of insecticide-treated nets were promoted and sold through different channels in the public and private sector, i.e. hospital pharmacy, mother and child health clinic, net committee, village health workers and retail shops. Two workers, one male and one female, were each given starting capital consisting of: five nets, enough insecticide for 200 net treatments, one set of treatment equipment, and a bicycle. In the first six months of the program, on average, the female worker bought 11 nets per supply trip and the male worker bought five nets totally 89 nets. The two workers were able to find customers, make sales to high-risk groups, provide health education, and generate income by selling the nets with relative ease. The research staff did indicate that it was more difficult to find the village health workers compared to institutional staff when delivering supplies. No information regarding the gender of individuals selling the insecticide-treated nets was given.
**Difficulties encountered**	There was not a high need for treatment of insecticide to nets. It was also noted that there was a lot of time needed to guide staff members.
**Study Recommendations**	The study suggests that a combination of private and public outlets can lead to the presence of availability and treatment of insecticide-treated bednets.

Khun S, Manderson L. Community participation and social engagement in the prevention and control of dengue fever in rural Cambodia. *Deng Bull.* 2008.

**Location of Study**	Kampong, Cambodia
**Human Development Index**	Medium
**Vector**	*Aedes aegypti*
**Disease**	Dengue
**Study Design**	Observational (inter-views, focus groups), cross-sectional
**Funding Agencies**	United Nations Children's Fund; United Nations Development Programme; World Bank; World Health Organization's Special Programme for Research and Research Training in Tropical Diseases
**Ethics Approval**	Human Research Ethics Committee of The University of Melbourne (Australia); Ministry of Health (Cambodia); World Health Organization's Special Programme for Research and Training in Tropical Diseases
**Study Quality**	Moderate
**Study Description**	Khun and colleagues explored how Cambodian villagers perceive community participation in dengue control, as well as the factors that influence community success in the prevention and control of dengue fever. Because women were perceived as being central players in dengue-related interventions, due to their domestic responsibilities, focus group discussions and in-depth interviews were conducted with women. Key Informant Interviews with staff from the local health centers, as well as from provincial and national levels offices were conducted. Due to years of political unrest, participants indicated that the collective spirit and sense of a community had declined in Cambodia. Therefore, villagers regarded the maintenance of their domestic environment as a personal responsibility and were reluctant to extend action outside of their compound unless payment was involved. Women reported that community based dengue control occurred, but, in practice, people had limited resources to participate in these activities. Women perceived dengue prevention and control to be a personal responsibility with few women reporting engagement in community level dengue control activities.
**Difficulties encountered**	Study subjects were reported to believe that community members were unaware and uninterested in the control of dengue because of the issue of poverty. Poverty caused the community's priorities to be more focused on obtaining needs such as food.
**Study Recommendations**	This study recommends that there be multisectoral collaboration as well as the utilization of local health facilities, important community members, and civil authorities. The study also suggests that welfare, engagement of the community, and community participation should be considered.

Supplement: Description of articles from database search

Kibe LW, Mbogo CM, Keating J, Molyneux S, Githure JI, Beier JC. Community based vector control in Malindi, Kenya. *Afr Health Sci*. 2006;6(4).*

**Location of Study**	Malindi, Kenya
**Human Development Index**	Low
**Vector**	*Anopheles* mosquito
**Disease**	Malaria
**Study Design**	Observational (inter-views, focus groups), cross sectional)
**Funding Agencies**	National Science Foundation; National Institutes of Health
**Ethics Approval**	None Reported
**Study Quality**	Moderate
**Study Description**	In Malindi, Kenya, community groups have become an integral part of the National Malaria Control Strategy. During key informant interviews, focus group discussions and stakeholder meetings, groups community groups that were involved with vector control were identified. The majority of the groups identified comprised of youth and women. One group - The Sheila Women Group - reported making and selling insecticide-treated nets. In addition to forming many of the community groups. Groups were provided with training and information, and disseminated materials and equipment to assist with project start-up. The Sheila Women's Group for example, reported receiving netting material, sewing machines, and thread to initiate insecticide-treated net development. Results indicate that community groups need clear government policies that outline the role of the government versus community members in terms of malaria control. Also, a supportive policy on incentives for participating community members and scientists should be developed as a next step for coordinating future integrated vector management programs in Malindi.
**Difficulties encountered**	A lack of continuous income was seen as a challenge for community group activities. Additionally, individuals' low financial status was reported to affect the involvement of group members.
**Study Recommendations**	There is a need to have an integrated program in which involves government workers, researchers, and community members in vector control. There should be more governmental support in financing efforts as well as creating clear policy which distinguishes roles of the government and the community.

* Part of the same research program as Mutero CM, Mbogo C, Mwangangi J, Imbahale S, Kibe L, Orindi B, et al. An Assessment of Participatory Integrated Vector Management for Malaria Control in Kenya. *Environ Health Presp*. 2015;123(11):1145.

Supplement: Description of articles from database search

Kovačič V. Women-led tsetse control: A pilot study in northwest Uganda. University of Liverpool; 2015.

**Location of Study**	North Western Uganda
**Human Development Index**	Low
**Vector**	Tsetse Fly
**Disease**	human African trypanosomiasis
**Study Design**	Participatory action/ Cohort
**Funding Agencies**	Bill and Melinda Gates Foundation
**Ethics Approval**	Research Ethics Committee of Liverpool School of Tropical Medicine; Uganda National Council of Science and Technology Ethics Committee
**Study Quality**	Moderate
**Study Description**	In Uganda, local women led a tsetse control program. Although the project had many aims, the primary woman focused aim was to pilot a women led tsetse control intervention using small pieces of cloth that were impregnated with insecticide, tiny targets, that attracted tsetse flies. Village A piloted an intervention where women where local women were enlisted to implement a tsetse control program through action research and Village B had an externally run program managed by team of "experts" and locally trained field technicians. Women mapped and measured the distance to local water sources in their communities. Women placed tiny targets that attracts tsetse flies around river beds 50 meters apart. Qualitative reports indicated that women noticed a reduction of flies at the water sources. Overall, women were more willing, motivated and organized to successfully manage operation. Their sense of ownership to the project and sense of empowerment increased which proved to be cost effective. Participants in Village A perceived the intervention as feasible. Because communities maintained the targets, more tiny targets were functional at six months post deployment in the pilot village than in an expert-led program in an adjacent area. At the end of the intervention, women requested replacement targets to deploy the following year.
**Difficulties encountered**	Women experienced challenges related to: time management, lack of tools to clear vegetation, lack of protective footwear, fear of snakes, and difficulties in accessing intervention areas. Women needed to be completed by noon due to weather and domestic tasks. Women reported that if provided with tools, protective footwear and some flexibility in hours, tsetse control interventions could be managed by women.
**Study Recommendations**	At the end of the intervention, women requested replacement targets to deploy the following year.

Supplement: Description of articles from database search

Kroeger A, Avinna A, Ordonnez-Gonzalez J, Escandon C. Community cooperatives and insecticide-treated materials for malaria control: a new experience in Latin America. *Malaria J*. 2002;1(15):1-15.

**Location of Study**	South Mexico and Pacific and Atlantic coasts of Colombia
**Human Development Index**	High
**Vector**	*Plasmodium vivax*, *Plasmodium falciparum*
**Disease**	Malaria
**Study Design**	Com-munity participatory methods
**Funding Agencies**	Department of International Development, Malaria-Knowledge Programme; European Commission
**Ethics Approval**	None Reported
**Study Quality**	Moderate
**Study Description**	Kroeger and colleagues discussed the process of creating 14 community-based cooperatives in Mexico and Columbia. Cooperatives offered specialized services that include the: production of nets(n=2); impregnation of existing nets (n=5); and impregnation of industrially produced or existing nets (n=8). Members of a prospective cooperative attended a training course about the insecticide-treated materials. At the start of the project, the research team donated provisions for impregnating roughly 1000 bednets. Any income obtained from the sales of the impregnated nets was put into a revolving fund for the purchase of future supplies and income for the cooperative members. Overall, six of the 14 cooperatives formed only contained female members. Each cooperative had elected officials, women held the majority of these positions within the cooperatives. Results of the project demonstrated that coverage of impregnated nets increased with low-cost cooperatives. With support and supervision by the health services, cooperatives have good potential to achieve and maintain high impregnation rates in the Latin American context.
**Difficulties encountered**	Direct supervision of the cooperatives was essential to program success; due to security reasons this could only occur inside larger areas. In Columbia, two cooperatives had funds stolen. Community members often didn't understand the need to re-impregnate mosquito nets or pay for it. Cooperatives in Columbia experienced higher insecticide costs due to an import tax.
**Study Recommendations**	Community cooperatives that impregnate material have good potential for decreasing vector borne diseases in Latin American.

Supplement: Description of articles from database search

Leygues M, Gouteux J-P. La lutte communautaire contre une endémie tropicale: croyances surnaturelles et pièges à tsétsé au Congo. *Soc Sci Med*. 1989;28(12):1255-1267.

**Location of Study**	Tambura County, South Sudan
**Human Development Index**	Low
**Vector**	Tsetse Fly
**Disease**	African trypanosomiasis
**Study Design**	Intervention/Observational (pre- and post-test)
**Funding Agencies**	Cooperative for Assistance and Relief Everywhere; International Medical Corps; United States Centers for Disease Control and Prevention
**Ethics Approval**	None Reported
**Study Quality**	Moderate
**Study Description**	In the South Sudan, an increase in African trypanosomiasis was experienced in the 1990s. The goal of this study was to increase community involvement in vector control of *Trypanosoma brucei* and to reduce the incidence of African trypanosomiasis. An integrated vector control approach was adopted by public health officials that focused on screening, treating, and training community-based vector control approaches in Tambura County, South Sudan. Between 1997-2001, 3250 traps were constructed and placed in high density areas by community volunteers. The average number of flies caught in each trap dropped significantly, from 25 to 3. The prevalence of trypanosomiasis was reduced from 9% to 2%. The success of the program was attributed to creating traps that were easily constructed from local materials, and were considered easy to set up, and maintain. Also, recruiting community volunteers from traditional birth attendants, and involving women's groups was described as crucial to the program success. Finally, the consultation and education of villagers before the intervention were imperative to raising the participation efforts.
**Difficulties encountered**	Not plausible to assume a causal relationship between community trapping activities and a reduction of disease prevalence.
**Study Recommendations**	Materials should be simple to make and use and community participants should be educated before an intervention to improve participation. Also, recruitment of community volunteers such as birth attendants and women's groups was seen as crucial to the intervention's success.

Supplement: Description of articles from database search

Makemba AM, Winch PJ, Kamazima SR, Makame VR, Sengo F, Lubega PB, et al. Community-based sale, distribution and insecticide impregnation of mosquito nets in Bagamoyo District, Tanzania. *Health Policy Plann*. 1995;10(1):50-59.

**Location of Study**	Bagamayo District, Tanzania
**Human Development Index**	Low
**Vector**	*Anopheles* mosquito
**Disease**	Malaria
**Study Design**	Intervention
**Funding Agencies**	United States Agency for International Development
**Ethics Approval**	None Reported
**Study Quality**	Moderate
**Study Description**	Designing, implementing and assessing the Bagamoyo Bednet Project occurred over a period of 5 years and was funded by the U.S. Agency for International Development, in Bagamoyo District of Tanzania. The project was designed to set up an insecticide-treated mosquito nets program with strong community participation, thereby measuring its impact on malaria transmission. It aimed at determining how the community could be involved through sustainable and successful implementation. The project aimed at addressing several key objectives related to both malaria control and local sustainability in the absence of external funding. The project area covered 13 villages, in which malaria transmission reduced and children's health improved considerably. The overall health of the community members had also improved and most of the people benefited from participation. The project staff developed effective messages that were communicated to community members by posters, skits, talks, and teaching through elected villagers. This resulted to a high level of participation and more people were willing to purchase bednets and insecticides. A system for accumulation of capital was developed within each village, and a management structure was set up and defined in a locally developed constitution.
**Difficulties encountered**	Low levels of malaria control coverage were caused by: dispersed population; inaccessible roads; high cost of vehicles and insecticides. Other challenges included actual costs of purchasing bednets, limited awareness of the health risks associated with malaria, few women participating in committees and limited knowledge on the roles and costs of insecticides.
**Study Recommendations**	The key to a sustainable community-based implementation plan involves building a partnership between the villagers, district authorities and the central government. It is necessary that the community acquires capital reserves in order to pay for services and supplies. This can be done by investing revenues obtained from sales of bednets and insecticides into village operated funds.

Supplement: Description of articles from database search

Mutero CM, Mbogo C, Mwangangi J, Imbahale S, Kibe L, Orindi B, et al. An Assessment of Participatory Integrated Vector Management for Malaria Control in Kenya. *Environ Health Presp*. 2015;123(11):1145.*

**Location of Study**	Malindi and Nyabondo, Kenya
**Human Development Index**	Low
**Vector**	*Anopheles* mosquito
**Disease**	Malaria
**Study Design**	Cohort
**Funding Agencies**	Biovision Foundation (Switzerland), International Centre of Insect Physiology and Ecology
**Ethics Approval**	None Reported
**Study Quality**	Moderate
**Study Description**	The study explored comprehensive strategies that effectively integrated both chemical and nonchemical methods of vector control into the existing healthcare system, so as to reduce the limitations associated with exclusive reliance on traditional vector control interventions such as the use of long lasting insecticidal nets and indoor residual spraying. The assessment of community based Integrated Vector Management (IVM) for malaria control in Kenya was carried out in two different sites; Nyabondo and Malindi regions, over a period of 6 years. Malindi, a coastal town and an urban setting, was a major tourist destination while Nyabondo, which is located on the west, near Lake Victoria, was mostly rural. The methods used in the study included qualitative external evaluation which assessed the IVM implementation process and its impacts on the community and other beneficiaries. This method employed institutional analysis that involved website review of the institutions that supported IVM as well as documentary analysis, which focused on interviewing project staff and community groups. The case data was collected by use of lights traps in both sites and pyrethrum spray catches in Malindi. Other methods that were used included evidence-based decision making, advocacy and social mobilizations, vertical and horizontal collaborations, integrated approaches, and capacity building. The results indicated that Malindi was more successful than Nyabondo in its IVM operations due to advantages of multi-stakeholder involvement as well as other supplementary resources it received from other donors. The community involvement through school and hospital based programs, women's groups, and malaria scouts (mostly women) helped to educate the public on malaria, thus leading to increased awareness and a reduced number of mosquito breeding sites. A number of the community members received training and eventually became employed due to participation in the project. The results showed there had been significant reduction in the number of malaria cases between 2009 and 2011 in Malindi, due to successful implementation of IVM.
**Difficulties encountered**	Barriers hindering optimal participation by the community in Nyabondo included the expectation of payment by some of the participants and the difficulty in determining if the brickmakers were only renting plots of land and were therefore not members of the community.
**Study Recommendations**	Awareness, involvement as well as horizontal and vertical collaborations between research institutes, policy makers and multi-stakeholders contribute more effectively to integrated vector management operations.

* Part of the same research program as Kibe LW, Mbogo CM, Keating J, Molyneux S, Githure JI, Beier JC. Community based vector control in Malindi, Kenya. *Afr Health Sci*. 2006;6(4).

Nading, A. M. Dengue mosquitoes are single mothers: Biopolitics meet ecological aesthetics in Nicaraguan community health work. *Cult Anthropol*. 2012; 27(4): 572-596.	
**Location of Study**	Nicaragua
**Human Development Index**	Medium
**Vector**	*Aedes aegypti*
**Disease**	Dengue
**Study Design**	Observational
**Funding Agencies**	Fulbright-Hayes Doctoral Dissertation Research Award; Social Science Research Council; National Science Foundation; University of Wisconsin-Madison Graduate Student Collaborative; Franklin & Marshall College.
**Ethics Approval**	None Reported
**Study Quality**	Low
**Study Description**	This observational study draws on 18 months of research in a Nicaraguan community. Community health workers, 90% of which were women, were taught how to identify, count, record and destroy foci within the community. Female participants indicated that women were better equipped to perform the role of vector control within the community as they had better social and negotiation skills and empathy than men. Women also indicated they had more intimate knowledge of the household.
**Difficulties encountered**	None reported
**Study Recommendations**	Creating a sense of detachment where community health workers were able to separate the person residing in the household, the environment and the mosquito was deemed essential to the program.

Pengvanich V. Family leader empowerment program using participatory learning process for dengue vector control. *J Med Assoc Thai*. 2011;94(2):235-241.

**Location of Study**	Chachoengsao, Thailand
**Human Development Index**	High
**Vector**	*Aedes aegypti*
**Disease**	Dengue Haemorrhagic Fever
**Study Design**	Quasi-experimental (pre- and post test)
**Funding Agencies**	None Reported
**Ethics Approval**	None Reported
**Study Quality**	Moderate
**Study Description**	A community empowerment program focused on controlling dengue utilized family leaders as executers of the program. Family leaders were provided knowledge about the prevention and control of dengue; approximately 70% of the family leaders were women. Family leaders were encouraged to assess the causes of the disease and problem solve how to control the Dengue vector on their own. Interviews and behavioral records of family leaders were collected, as well as counts of breeding containers and aedes larvae surrounding the home. After participating in the program for eight weeks, A significant reduction in the *Aedes aegypti* was demonstrated in both the container index and the house index post intervention.
**Difficulties encountered**	Several family leaders were not able to attend the 2-day workshop. Others did not sufficiently understand how to fill out the aedes larval survey form.
**Study Recommendations**	Vector control should incorporate cooperation and collaboration of family leaders, public health personnel, and public health volunteers.

Sharma R, Gautam A, Bhatt R, Gupta D. Community participation and intersectoral cooperation in malaria control in Kheda District, Gujarat. *Comm Partic Malaria Control.* 1993:123-132.

**Location of Study**	Nadiad Taluka of Kheda District, Gujarat, India
**Human Development Index**	Medium
**Vector**	*Anopheles* mosquito
**Disease**	Malaria
**Study Design**	Community participatory methods
**Funding Agencies**	None Reported
**Ethics Approval**	None Reported
**Study Quality**	High
**Study Description**	The study focused on bioenvironmental malaria control strategy through community participation and intersectoral cooperation. It was implemented over a period of 5 years covering 100 villages. The main methods used in the malaria control program included health education and community participation. Health educators offered demonstrations and exhibitions on the life cycles of malaria parasites using charts and microscopes. Women health educators engaged in door to door visits in the different villages providing health education, tree planting and village sanitation activities. Community participation was emphasized through formation of community groups in which discussions about malaria were held and led by project staff. Other initiatives included erection of health camps, voluntary labor camps, and breeding source management activities. The community also benefited from the project through environmental improvement, incentives and other income generating schemes. Cooperatives and other local voluntary organizations contributed actively to the control program through provision of technical guidance and motivation to the community members. Successful control measures ensured low density of malaria parasites throughout the study period in both inter and intra-domestic sources. The two surveys conducted at the end of the study revealed that 82% of the villagers viewed the program as successful in creating awareness and sustainable malaria control methods in their village.
**Difficulties encountered**	There were difficulties in implementing certain control activities such as introducing larvivorous fish in intra-domestic breeding sources or emptying them.
**Study Recommendations**	All the project staff working in laboratories and field were not trained. It is important to include trained health educators in future control programs.

Supplement: Description of articles from database search

Silva K, Navaratna H, Rao MA, Wanninayaka P, Doolwala S, Karunaratna N, et al. Malaria control through community action at the grass-roots: experience of the Sarvodaya malaria control research project in Sri Lanka from 1980 to 1986. Available from: http://apps.who.int/iris/handle/10665/58725

**Location of Study**	Anuradhapura District, Sri Lanka
**Human Development Index**	High
**Vector**	*Anopheles* mosquito
**Disease**	Malaria
**Study Design**	Observational
**Funding Agencies**	World Health Organization; World Bank; United Nations Development Programme; World Health Organization’s Special Programme for Research and Training in Tropical Diseases
**Ethics Approval**	None Reported
**Study Quality**	Moderate
**Study Description**	The study was conducted in Anuradhapura District of Sri Lanka, to provide malaria control through community participation. Some of the objectives of the project included helping villagers identify the malaria vector and its annual cycle, to bring malaria control at the village level, to expand community based surveillance systems to cover all illnesses and to determine the management capacity of a non-governmental organization in implementing community based primary health care. The project used control interventions such as filling and draining of vector breeding sites and introduction of larvivorous fish to predate on mosquito larvae on ponds. Methods used for data collection such as parasite surveillance, vector surveillance and geographical reconnaissance, involved trained workers at the village level with the help of other community members. Case data of the three vector control interventions showing the number of adult *Anopheles* collected through the standard tests per 1000 population, was used to assess the impact of the surveillance program. The results indicated that the successful implementation of the malaria control program was due to factors such as surveillance through community action, development of a primary healthcare approach to malaria control, prevention and control of malaria epidemics, intersectoral collaborations and non-governmental organization involvement. Women played a crucial role in the project at the village level as coordinators, school workers, health workers and community organizers.
**Difficulties encountered**	Difficulty in developing alert mechanisms through which the community members could continuously be alerted about the possibility of recurrence of malaria epidemics.
**Study Recommendations**	It is necessary to refine and develop surveillance and malaria containment strategies used in the Sarvodaya Malaria Control Research Project. Vector biology component should be strengthened with inputs from various collaborating institutions such as anti-malaria campaign.

Sindato C, Kimbita E, Kiboma S. Factors influencing individual and community participation in the control of tsetse flies and human African trypanosomiasis in Urambo District, Tanzania. *Tanzan J Health Res*. 2008;10(1):20-27.

**Location of Study**	Urambo, Tanzania
**Human Development Index**	Low
**Vector**	Tsetse fly
**Disease**	human African trypanosomiasis
**Study Design**	Cross-sectional
**Funding Agencies**	National Institute for Medical Research, Tanzania
**Ethics Approval**	National Institute for Medical Research, Tanzania
**Study Quality**	Moderate
**Study Description**	This study involved community participation in tsetse fly and human African trypanosomiasis (HAT) disease control activities in Urambo district, located in western Tanzania. Insecticide impregnated targets and traps were actively used, and community involvement was relied upon in the control program. Nine villages were selected and Semi-structured questionnaires were used to collect information from individuals in randomly selected households. More data was obtained from the medical records of Kaliua Health Centre and Urambo District Hospital. The results of the study indicated that knowledge of tsetse flies and HAT were key factors in influencing the community's contribution of resources and participation in the vector control program. The study shows that more men (92.9%) participated in the HAT control activities than women (7.1%) by contributing money and labor. Reports also indicated that men were perceived to be stronger (40%) and earned more money than women, but were more likely to suffer from the disease. There were limited opportunities for women (24.7%) to participate in HAT control activities in Urambo district. Despite having knowledge of tsetse flies, the community members barely understood the roles of animals in HAT epidemiology.
**Difficulties encountered**	Theft and vandalism of traps and targets is a major challenge.
**Study Recommendations**	Creating local awareness on tsetse fly and trypanosomiasis problems, and use of suitable technology to help reduce theft and vandalism. The community or extension staff should be involved in the initial design of the control programs.

Sleigh A, Jackson S, Huang K. Eradication of schistosomiasis in Guangxi, China. Part 2: Political economy, management strategy and costs, 1953-92. *B World Health Organ*. 1998;76(5):497-508.

**Location of Study**	Guangxi, China
**Human Development Index**	High
**Vector**	Snail
**Disease**	Schistosomiasis
**Study Design**	Observational
**Funding Agencies**	World Health Organization; World Bank; United Nations Development Programme; World Health Organization's Special Programme for Research and Training in Tropical Diseases
**Ethics Approval**	None Reported
**Study Quality**	Low
**Study Description**	Guangxi Schistosomiasis Control Program was established by the Chinese government between 1953 and 1992. As part of the control program, epidemiological surveillance which aimed at eradicating the disease was initiated through the Guangxi Institute for Parasitic disease control. Other multi-sectoral advisory bodies, scientific research institutes and special hospitals were also involved. The methods used included postal questionnaires and participation of farmers, teachers, office workers, soldiers, and students through mass campaigns and involvement in activities such as scraping earth, piling up compost, filling up gullies, transforming low-lying land and burning materials in which snails breed. They also dug new ditches while filling old ones, tunneled through mountains to drain waterlogged lands, built storage dams and reclaimed wasteland. The control program boosted rural production by transforming the snail breeding environments into agriculturally productive sites. Applied research on geographical, agricultural, behavioral, and ecological facets of transmission, as well as local features of infection and disease led to successful implementation of the control activities. Multi-sectoral and other leading groups such as public health, agriculture, Youth League, and the Women's Federation made the elimination process a priority on their local agenda. This particular approach also contributed to the programs financially and administratively in collaboration with the ministry of health and the central Chinese government.
**Difficulties encountered**	The eradication program faced challenges in the 1960s, a period of both political and economic turmoil. This slowed down the control program.
**Study Recommendations**	Government involvement is very critical in the success of control programs. Multisectoral collaboration is also key to successful implementation of control programs.

Supplement: Description of articles from database search

Sommerfeld J, Kroeger A. Eco-bio-social research on dengue in Asia: a multicountry study on ecosystem and community-based approaches for the control of dengue vectors in urban and peri-urban Asia. *Pathog Glob Health*. 2012;106(8):428-435.*

**Location of Study**	Chennai, India; Yogyakarta, Indonesia; Yangon, Myanmar; Muntinlupa, Philippines; Gampaha district, Sri Lanka; Chachoengsao province, Thailand
**Human Development Index**	High: Sri Lanka, Thailand;<br>Medium: India, Indonesia, Philippines;<br>Low: Myanmar
**Vector**	*Aedes aegypti*
**Disease**	Dengue
**Study Design**	Cohort
**Funding Agencies**	International Development Research Centre of Canada
**Ethics Approval**	None Reported
**Study Quality**	High
**Study Description**	Sommerfeld and colleagues conducted an interdisciplinary investigation of ecological, biological, and social (i.e., eco-bio-social) dimensions of dengue in urban and peri-urban areas in India, Sri Lanka, Indonesia, Myanmar, Philippines and Thailand. The common goals across the study sites were to: (1) design, conduct and evaluate multi-partnership interventions with emphasis on community involvement; and to (2) assess the effect of the intervention on partners and the vector populations. By developing community-based interventions aimed at reducing dengue vector breeding and viral transmission the study site mobilized and empowered women's group as well as student and community groups to decrease vector density. Strong involvement of women in the intervention were reported in all but the Philippines. Although the article reports that women actively participated in the project, authors also acknowledge that women played a minor role in decision-making. Only information regarding women's involvement in India was included in this article. In India, women were instrumental in delivering non-insecticidal water container covers for cement tanks, drums and barrels. Overall, peoples' knowledge of dengue transmitting mosquitoes was associated with reduced mosquito breeding and production, attributed to increased self-protection with domestic insecticides. Vector control measures substantially reduced the larval/pupal indices and 'pushed' mosquito breeding to alternative containers. The programs led to the formation of community groups and other public and private partners.
**Difficulties encountered**	None reported
**Study Recommendations**	In order for vector management to be sustainable it should only complement or replace other interventions by: (1) involving diverse partners — including local communities, (2) targeting water container interventions that achieve a significant reduction of dengue vectors, and (3) utilizing novel non-insecticidal intervention tools.

* Part of the same research program as Arunachalam N, Tyagi BK, Samuel M, Krishnamoorthi R, Manavalan R, Tewari SC, et al. Community-based control of *Aedes aegypti* by adoption of eco-health methods in Chennai City, India. *Pathog Glob Health*. 2012;106(8):488-496; Espino F, Marco J, Salazar NP, Salazar F, Mendoza Y, Velazco A. Community-based dengue vector control: experiences in behavior change in Metropolitan Manila, Philippines. *Pathog Glob Health*. 2012;106(8):455-460; Tana S, Umniyati S, Petzold M, Kroeger A, Sommerfeld J. Building and analyzing an innovative community-centered dengue-ecosystem management intervention in Yogyakarta, Indonesia. *Pathog Glob Health*. 2012;106(8):469-478.

Suroso T. *Aedes aegypti*, control through source reduction by community efforts in Pekalongan, Indonesia. *Mosq Borne Dis Bull*. 1990;7(2):59-62.

**Location of Study**	Pekalongan, Indonesia
**Human Development Index**	Medium
**Vector**	*Aedes aegypti*
**Disease**	Dengue Haemorrhagic Fever
**Study Design**	Observational
**Funding Agencies**	Directorate General of Disease Control and Environmental Health; Ministry of Health
**Ethics Approval**	None Reported
**Study Quality**	Moderate
**Study Description**	An intervention of health education by community efforts via source reduction was executed in Pekalongon, Indonesia. Source reduction involved clearing mosquito breeding habits as well as health education. The intervention reached a total of 599 buildings, including 133 schools, 266 school children's dwellings, and 200 other houses. The local Women's Club was used to lead a source reduction campaign in 1985. The roles of these individuals were to give health education and to inspect mosquito larva. The volunteers were responsible for 150 buildings and were expected to visit homes once a month. Six months after the initiation of the intervention it was determined that premise, container, and Breteau indices were reduced. A limitation is that there was no control arm of the intervention. In conclusion, it was determined that reduction of the aforementioned indices were greater in other households rather than schools or dwellings of school children. It was stated that there is a need for greater efforts to target children as they make up a large proportion of the population in Indonesia.
**Difficulties encountered**	There was no control area in the intervention. Additionally, it was mentioned that the control measure of source reduction was difficult for the case of water storage containers, flower vases, and ant traps. Moreover, children were believed to be less motivated to participate in the control measure activities that the intervention aimed for.
**Study Recommendations**	Source reduction and community efforts can be utilized for a reduction in the premise, container, and Breteau indices. There should be consideration to target school children during the intervention because of the large proportion of youth in the Java population.

Supplement: Description of articles from database search

Tana S, Umniyati S, Petzold M, Kroeger A, Sommerfeld J. Building and analyzing an innovative community-centered dengue-ecosystem management intervention in Yogyakarta, Indonesia. *Pathog Glob Health*. 2012;106(8):469-478.*

**Location of Study**	Yogyakarta city, Indonesia
**Human Development Index**	Medium
**Vector**	*Aedes aegypti*
**Disease**	Dengue
**Study Design**	Cohort
**Funding Agencies**	United Nations Children's Fund; United Nations Development Programme; World Bank; World Health Organization’s Special Programme for Research and Training in Tropical Diseases; International Development Research Centre of Canada
**Ethics Approval**	Ethical Review Committee of the World Health Organization; Ethical review board in Yogyakarta
**Study Quality**	High
**Study Description**	In collaboration with Sommerfeld and colleagues, Tana and colleagues also conducted an interdisciplinary investigation of ecological, biological, and social (i.e., eco-bio-social) dimensions of dengue in urban and peri-urban areas in Indonesia. Women actively participated via women’s groups and were instrumental in decision making in Indonesia. Women's role was to partner in educating other family members; they were decision makers for intervention at the family level, particularly in relation to women’s roles in the family, including water management. No other information regarding the role of women in the study was provided.
**Difficulties encountered**	Starting a community centered dengue control program required a lot of time and energy at the beginning of the program but can be sustained over time.
**Study Recommendations**	Engaging the community to contribute either financially or with in-kind support increases the sustainability of the projects.

* Part of the same research program as Arunachalam N, Tyagi BK, Samuel M, Krishnamoorthi R, Manavalan R, Tewari SC, et al. Community-based control of *Aedes aegypti* by adoption of eco-health methods in Chennai City, India. *Pathog Glob Health*. 2012;106(8):488-496; Espino F, Marco J, Salazar NP, Salazar F, Mendoza Y, Velazco A. Community-based dengue vector control: experiences in behavior change in Metropolitan Manila, Philippines. *Pathog Glob Health*. 2012;106(8):455-460; Sommerfeld J, Kroeger A. Eco-bio-social research on dengue in Asia: a multicountry study on ecosystem and community-based approaches for the control of dengue vectors in urban and peri-urban Asia. *Pathog Glob Health*. 2012;106(8):428-435.
